# Spontaneous Hemoperitoneum in Pregnancy: Masquerading as Acute Appendicitis

**DOI:** 10.7759/cureus.47040

**Published:** 2023-10-14

**Authors:** Johnbosco Mamah, Megan Thomas, Junaid Rafi

**Affiliations:** 1 Obstetrics and Gynaecology, East Suffolk and North Essex National Health Service (NHS) Foundation Trust, Ipswich, GBR

**Keywords:** emergency laparotomy, spontaneous hemoperitoneum, : pregnancy, severe endometriosis, s: acute appendicitis

## Abstract

Spontaneous hemoperitoneum in pregnancy (SHiP) is a rare obstetric emergency that may have an adverse outcome for the mother and baby. This case report describes a unique SHiP case initially diagnosed as acute appendicitis in a patient with severe endometriosis before conception. A woman in her 30s, a primigravida, was admitted with abdominal pain at 32+5 weeks gestational age. Following a surgical review, she was initially diagnosed with acute appendicitis and commenced on intravenous antibiotics. She experienced a dramatic deterioration in her health in the form of clinical shock and fetal distress. She had an emergency laparotomy, a hysterectomy, and a left salpingo-oophorectomy for uncontrollable bleeding. The baby was born in good health, and the mother had an uneventful recovery.

## Introduction

Spontaneous hemoperitoneum during pregnancy is rare [1,2]. It is characterized by spontaneous intraperitoneal bleeding during pregnancy and up to 42 days after delivery, necessitating surgical intervention [1-3]. SHiP excludes cases involving uterine rupture, ruptured ectopic pregnancies, and bleeding related to cesarean sections [2,3]. SHiP has been reported in various contexts, including ruptures of the splenic artery, uterine arteries, varicose veins, or aneurysms. However, a growing body of evidence points to endometriosis as a significant contributing factor [3-10]. SHiP in a patient with a history of endometriosis may result from the rupture of endometriomas, bleeding from utero-ovarian vessels, or hemorrhage originating from endometriotic implants eroding pelvic blood vessels [4-6].

## Case presentation

A primigravida woman in her 30s presented at 32+5 weeks of gestation with a sudden onset of continuous abdominal pain. The pain was dull and most pronounced in the left lower abdomen. There was no vaginal loss, fever, nausea, vomiting, or urinary tract symptoms. The pain was exacerbated by physical activity and relieved temporarily by analgesia. On presentation, her vital signs were within the normal range, and a urinalysis showed no abnormalities. Abdominal examination revealed diffuse tenderness with guarding. There was no palpable uterine contraction. Fetal monitoring with a cardiotocograph (CTG) was normal. She was admitted and given regular pain relief. The patient initiated antenatal care at our facility at 27 weeks of gestation. She had a significant medical history of severe endometriosis and had undergone laparoscopic cystectomy for a ruptured left endometrioma before conceiving while being worked up for in-vitro fertilization.

**Figure 1:**
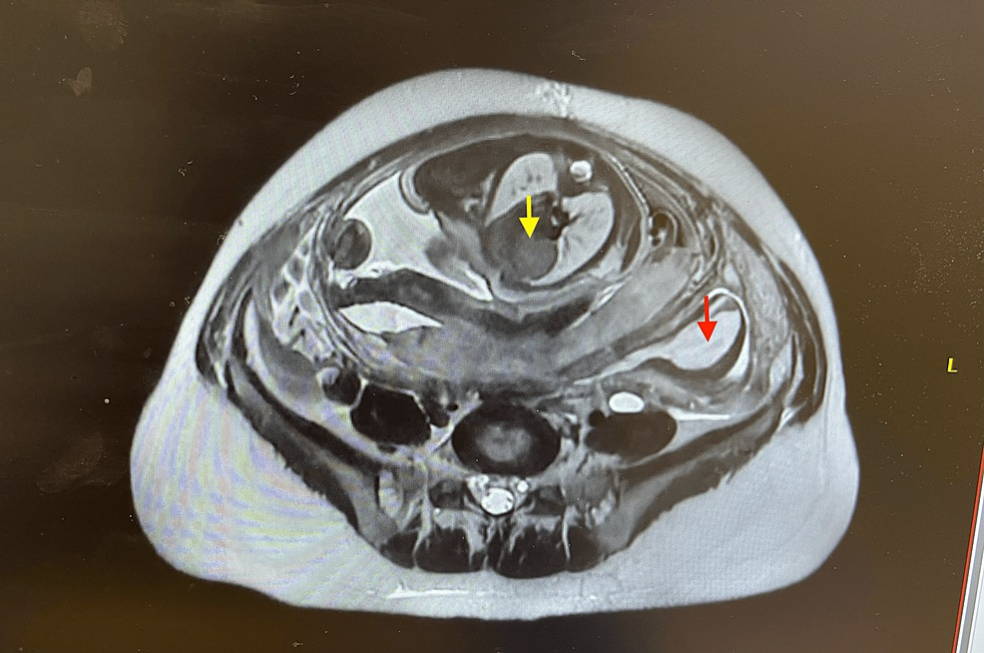
Transverse view MRI of the abdomen and pelvis. The image describes a transverse view magnetic resonance image (MRI) of the patient's pelvis showing a complex left adnexal cyst (marked with a red arrow) adjacent to the uterus, which harbors the fetus, marked with a yellow arrow.

**Figure 2:**
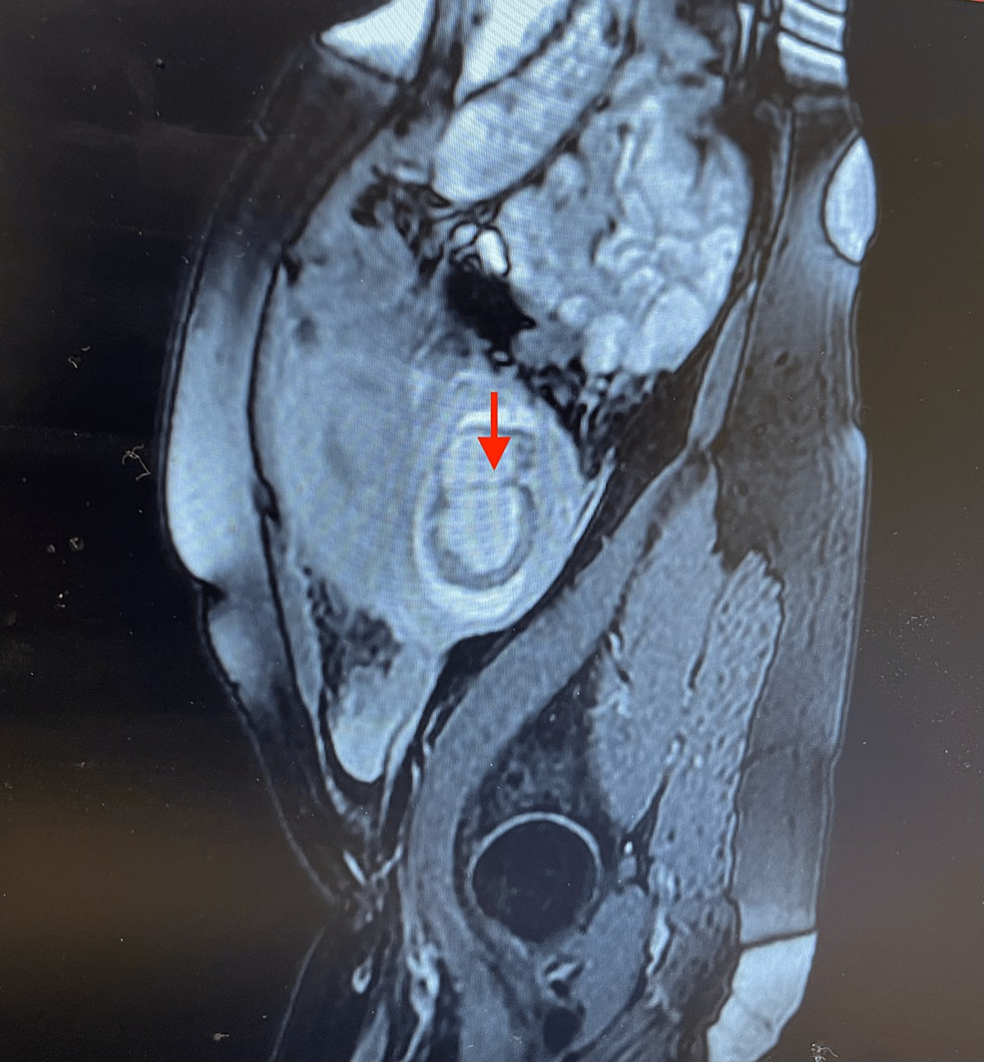
A sagittal-plane MRI of the abdomen and pelvis. The image describes sagittal plane magnetic resonance imaging (MRI) showing a complex cystic left adnexal mass adhering to the uterus (marked with a red arrow).

**Figure 3:**
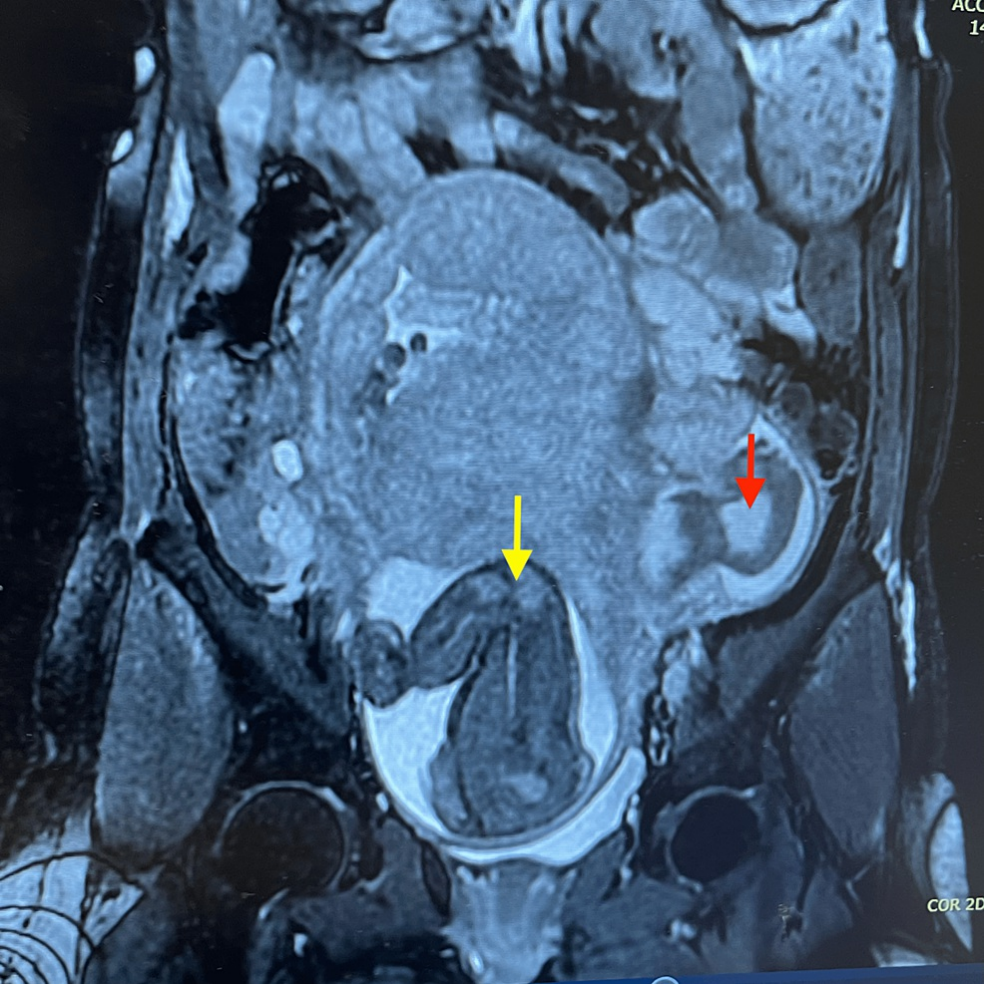
Coronal view MRI showing the abdomen and pelvis. The image shows a preoperative plane magnetic resonance image (MRI) (anterior-posterior view) of the abdomen and pelvis, showing a gravid uterus with a breech fetus (marked with a yellow arrow) and a left complex adnexal lesion adherent to the uterus and the pelvic side wall (marked with the red arrow).

**Figure 4:**
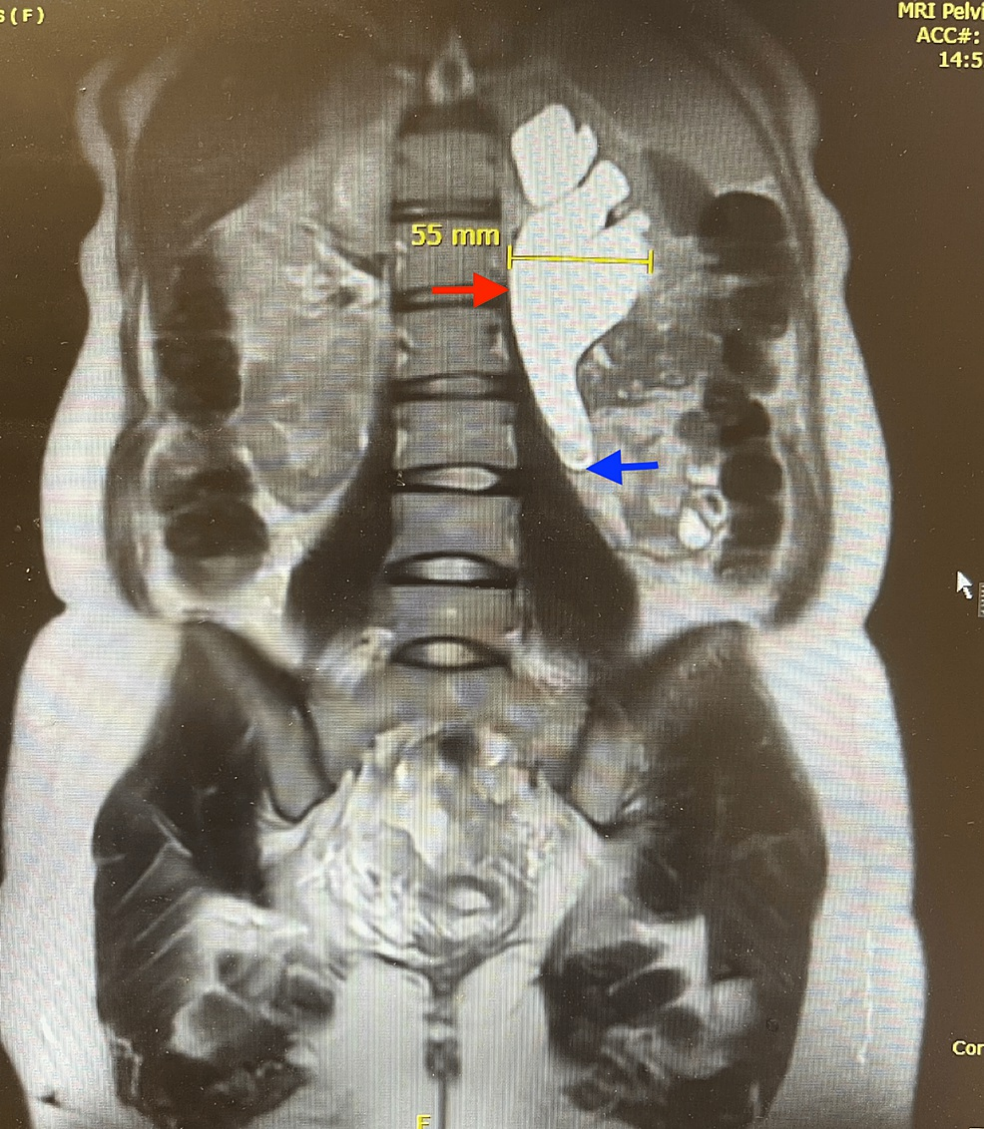
Postoperative MRI shows severe left hydronephrosis. The image shows coronal plane magnetic resonance imaging (MRI) showing severe left hydronephrosis measuring 55 mm (marked with the red arrow) caused by a distal ureteric obstruction from an endometriotic lesion at the point where the ureter abruptly ends (marked with the blue arrow).

Her blood investigations revealed a hemoglobin level of 111g/L, a white blood cell count of 20.1 x 10^9 /L, and neutrophilia at 18.1 x 10^9 /L. The C-reactive protein was 11mg/L. The venous lactate was 1.2mmol/L. Renal and liver function tests were within the normal range. An urgent abdominopelvic ultrasound revealed a bilateral hydronephrosis and a small pelvic fluid collection, but the appendix was not visualized. Without an obstetric cause for her pain, the acute surgical team was invited to review the patient. Upon review by the surgical team, a provisional diagnosis of acute appendicitis was made. Intravenous antibiotics and fluid management were initiated. A pelvic magnetic resonance imaging (MRI) scan was ordered; it found a left complex tubo-ovarian lesion measuring approximately 5.0 x 3.5 cm, displaying characteristics consistent with a hemorrhagic ovarian cyst, possibly of endometriotic origin (Figures 1-3); there was severe hydronephrosis of the left ureter (Figure 4); and the appendix was reported as normal. Antibiotics and surgical inputs were discontinued. Due to her ongoing pain, she remained admitted for pain management and observation. A repeat full blood count conducted 24 hours later revealed a decline in hemoglobin levels, with a reading of 90 g/L. This drop in hemoglobin from her baseline of 111g/L raised the possibility of ongoing intra-abdominal bleeding from the hemorrhagic ovarian cyst reported on the MRI. However, she was clinically stable, had normal vital signs, and reported improved symptoms. About 36 hours into hospital admission, she woke up with sudden, severe abdominal pain and light-headedness. She sought assistance from the medical staff. Her vital signs showed a blood pressure of 60/40 mmHg and a heart rate of 115 beats per minute. Upon examination, she was pale and had severe abdominal tenderness, and the uterus was soft. There was no vaginal bleeding. The fetal CTG was pathological. Venous blood gas analysis revealed serum lactate level of 3.3mmol/L and a hemoglobin level of 79 g/l. A diagnosis of intraperitoneal bleeding was made. Immediate fluid resuscitation was initiated, and blood products were requested. She consented and was taken for an emergency laparotomy under general anesthesia. Intraoperatively, we found 2.5 liters of hemoperitoneum with active bleeding from a left tubo-ovarian complex. A lower segment uterine incision was performed, and a live female baby was delivered with an APGAR score of one in the first minute and eight in the fifth minute. The uterus, adnexa, and upper abdomen were examined. A complex tubo-ovarian structure (suspected endometrioma) measuring approximately 8 cm on the left adnexa was found intimately attached to the uterus and the left pelvic wall with ill-defined anatomical planes. The lesion appeared necrotic and had ruptured, resulting in active arterial bleeding. The presence of an old hematoma in the Pouch of Douglas (POD) and sub-hepatic recesses indicated possible ongoing slow bleeding. The right adnexa was grossly normal. Attempts at sequential devascularisation of the left adnexa were unsuccessful, necessitating a left salpingo-oophorectomy. The patient continued to bleed actively, and a sub-total hysterectomy was performed, which led to the cessation of the bleeding. The total measured blood loss was 4.7 liters. The patient received transfusions of five packed red blood cells, four fresh frozen plasma (FFP) units, and one platelet pool. Histological examination of the excised left tube and ovary showed the presence of 'decidualized endometrial glands with Arias-Stella reaction,' thus confirming the diagnosis of endometriosis/endometrioma. The left ovary displayed a partial infarction.

The patient subsequently made an uncomplicated recovery and was discharged on the fifth day after surgery. After a few weeks of neonatal care, the baby was discharged from the neonatal unit. During her six-week postnatal follow-up visit, repeat magnetic resonance imaging (MRI) indicated the persistent presence of left hydronephrosis and cortical thinning resulting from distal ureteric obstruction caused by endometriosis.

## Discussion

The exact incidence of this condition in patients with severe endometriosis remains unknown, but an estimate of one in 10,000 has been reported [11]. The incidence will rise as more women with endometriosis, previously considered sub-fertile, conceive through in vitro fertilization (IVF) [5]. A national survey commissioned by the United Kingdom Obstetric Surveillance System (UKOSS) in 2016 is underway to determine the incidence of SHiP in the UK, with results expected to be published soon [3]. The underlying cause is not fully understood; rupture of endometriotic cysts, decidual hemorrhage, and hormonal and vascular changes associated with pregnancy have been implicated [2-16]. There is a higher incidence of SHiP in the second half of pregnancy, possibly because, during pregnancy, the uterus experiences increased blood supply, leading to physiological hypertrophy of utero-ovarian vessels. Chronic inflammation of endometriotic lesions renders surrounding tissues and vessels fragile. As the uterus enlarges, adhesions create traction on surrounding tissue, making the affected tissue or vessels susceptible to bleeding as the uterus rapidly grows in the second and third trimesters [4-18].

SHiP typically presents with acute-onset abdominal pain [1-20], often accompanied by signs of hemodynamic instability. Patients may exhibit diffuse abdominal tenderness, guarding, and rebound tenderness, which can lead to misdiagnosis and delayed treatment [4-10]. Some patients also present with signs of fetal distress detected on CTG or ultrasound scans. Evidence suggests a higher incidence of SHiP arising from endometriosis affecting the left adnexa than the right [6,9,13], although no clear explanation exists. The two cases reported from our unit, including the present case, involved the left hemipelvis [6].

Diagnosing spontaneous hemoperitoneum in pregnancy presents a significant challenge due to the nonspecific clinical presentation [1-20] and symptom overlap with placental abruption and uterine rupture. Diagnostic modalities such as ultrasound, MRI, and CT abdominopelvic scans will exclude other surgical causes. Surgical intervention, typically involving laparotomy, is often required to identify and control the source of bleeding [1-20].

The decision regarding fetal delivery should be based on the clinical situation. Among the cases reviewed in the literature (Table 1), only one patient had a laparotomy and carried her pregnancy to term [18]. Maternal mortality due to SHiP has significantly decreased, but fetal mortality remains high (Table 1). 

**Table 1:** A literature review of some SHiP cases in pregnant patients was reported in 2016-2023. The table illustrates some recent case reports of spontaneous hemoperitoneum in pregnancy, including the maternal and fetal outcomes. CTG: Cardiotocograph, CT scan: Computerised tomography scan, FFP: Fresh frozen plasma, IVF: In-vitro fertilisation, ML: Milliliter, MRI: Magnetic resonance imaging.

Author	Gestation at presentation	Case history	Imaging at diagnosis	Risk factors	Management approach	Blood loss	Outcome
Bazzurini et al., 2023 [1].	Between 26 and 37 weeks of gestation. One was postpartum.	All had abdominal pain. Mean maternal age of 34 years. Two had abnormal CTG.	A combination of an MRI and pelvic ultrasound confirmed free fluid.	Three are known to have severe endometriosis.	Four had emergency laparotomies, and two managed conservatively. Haemostasis was achieved with suture ligation of bleeding vessels.	Average blood loss: 4250 ml.	All the patient and their babies did well.
Kim et al., 2020 [4].	Six weeks of gestation.	36-year-old with acute abdominal pain and vaginal bleeding. It was misdiagnosed as a ruptured ectopic pregnancy.	Pelvic ultrasound.	Endometriosis	Laparoscopic electrocoagulation of bleeding vessels.	1800 ml.	She suffered a miscarriage after surgery.
Aliyu et al., 2021 [5].	24 weeks.	Abdominal pain and dizziness. Known endometriosis, conceived with twin pregnancy with IVF. Initially managed as non-specific abdominal pain and discharged.	Pelvic ultrasound shows free fluid.	Endometriosis and IVF.	Laparotomy, hysterectomy, and right adnexectomy, and she also received 19 units of red cells, 15 FFP, eight pools of platelets, and two cryoprecipitates.	5000 ml	The mother did well, but the babies died shortly after delivery.
Huang et al., 2021 [10].	18 weeks.	35-year-old misdiagnosed with peritonitis, then sudden maternal shock and fetal demise.	A pelvic ultrasound scan shows free fluid.	Endometriosis.	Emergency laparotomy with suture ligation of bleeding vessels.	1500 ml.	The mother did well, and the baby died.
Gomez et al., 2022 [13].	35 weeks.	Severe abdominal pain and fetal distress.	Pelvic ultrasound.	Known endometriosis.	Cesarean section and suture ligation of bleeding vessels.	2000 ml.	Mother and baby did well.
Sim et al., 2020 [14].	27 weeks.	31-year-old with severe abdominal pain and shock initially managed for non-specific abdominal pain.	Pelvic ultrasound scan and confirmed on MRI showing hemoperitoneum.	Known as severe endometriosis.	Laparotomy and suture ligation of bleeding vessels.	2000 ml.	Mother and baby did well.
Author	Gestation at presentation	Case history	Imaging at diagnosis	Risk factors	Management approach	Blood loss	Outcome
Kim et al., 2020 [15].	27 weeks.	38-year-old Primip with vague abdominal pain.	Pelvic ultrasound scan.	Known endometriosis.	Emergency cesarean hysterectomy and bilateral salpingo-oophorectomy due to massive bleeding.	6000 ml.	Mother and baby did well.
Da Silva et al., 2020 [16].	22 weeks.	32-year-old Primip presented with severe abdominal pain and shock.	Pelvic ultrasound scan and CT of abdomen and pelvis showing hemoperitoneum.	Spontaneous vessel rupture.	Emergency laparotomy and suture ligation.	2000 ml.	The mother did well but suffered a miscarriage four days postop with fetal demise.
Vuong et al., 2023 [17].	22 weeks and 35 weeks, respectively.	Abdominal pain and should be accompanied by fetal distress.	A pelvic ultrasound scan shows free fluid.	Ruptured endometrioma and bleeding from scar tissue, respectively.	Emergency laparotomy and cesarean section.	2000 ml.	The mothers did well, but there was a spontaneous miscarriage for the 22-week patient and a live healthy birth for the 35-week patient.
Jang et al., 2016 [18].	30 weeks.	A 27-year-old para 1 presenting with abdominal pain.	Pelvic ultrasound scan showing hemoperitoneum.	A spontaneously ruptured uterine vessel.	Emergency laparotomy and suture ligation.	2000 ml.	The mother recovered and had a live birth at term via planned cesarean section.
Markou et al., 2017 [19].	Third trimester.	30-year-old presenting with severe abdominal pain.	Pelvic ultrasound scan showing hemoperitoneum.	Spontaneous rupture of pelvic vessels.	Emergency cesarean section.	3000 ml and 4000 ml.	Mothers and their babies did well.
Kato et al., 2022 [20].	28 weeks.	41-year-old presenting with recurrent abdominal pain, initially misdiagnosed as a pelvic infection.	Pelvic ultrasound scan showing hemoperitoneum.	Known endometriosis and conceived by IVF.	Emergency cesarean section and ligation of bleeding vessels.	2000 ml.	Mother and baby did well.

## Conclusions

This case report sheds light on a challenging clinical scenario: spontaneous hemoperitoneum during pregnancy at 32 weeks of gestational age, compounded by severe endometriosis. This rare and high-risk combination presented a complex clinical picture, leading to massive intraperitoneal bleeding and necessitating an emergency premature delivery. The case underscores the importance of recognizing and closely monitoring pregnant patients with known severe endometriosis, as they are at an increased risk for such complications. Early diagnosis and swift intervention are paramount in preventing maternal and fetal morbidity and mortality. We further emphasize the significance of a multidisciplinary approach involving obstetricians, surgeons, and specialists in endometriosis management. Collaboration and timely decision-making were essential to achieving a successful outcome in this case. It also highlights the need for further research to understand the intricate relationship between the pathogenesis of endometriosis and spontaneous hemoperitoneum during pregnancy, aiming to improve prevention and management strategies to achieve good maternal and fetal outcomes. Ultimately, successful outcomes in such cases rely on the collaborative efforts of the healthcare team and the ability to adapt swiftly to emergent situations, ensuring the well-being of both mother and child.
